# The “island sign” on diffusion-weighted imaging predicts early neurological deterioration in penetrating artery territory infarctions: a retrospective study

**DOI:** 10.1186/s12883-023-03351-y

**Published:** 2023-08-11

**Authors:** Yang Yang, Zhihua Si, Dawei Wang, Dong Dong, Rutao Liu, Xianwen Yu, Jiyou Tang, Aihua Wang

**Affiliations:** 1 Department of Neurology, Shandong Provincial Qianfoshan Hospital, Shandong University, No. 16766 Jingshi Road, Jinan, 250013 Shandong China; 2 Department of Neurology, Shandong Key Laboratory of Rheumatic Disease and Translational Medicine, The First Affiliated Hospital of Shandong First Medical University and Shandong Provincial Qianfoshan Hospital, Shandong Institute of Neuroimmunology, No. 16766 Jingshi Road, Jinan, 250013 Shandong China; 3 Department of Radiology, The First Affiliated Hospital of Shandong First Medical University and Shandong Provincial Qianfoshan Hospital, No. 16766 Jingshi Road, Jinan, 250013 Shandong China

**Keywords:** Penetrating artery territory infarction, Island sign, Early neurological deterioration, Lenticulostriate arteries, Paramedian pontine arteries, Diffusion-weighted imaging(DWI)

## Abstract

**Background:**

Early neurological deterioration (END) sometimes occurs in patients with penetrating artery territory infarction (PATI) and leads to poor prognosis. In this study, we analyzed clinical and neuroimaging characteristics of PATI, and focused on the infarct patterns on diffusion-weighted imaging (DWI). We tried to investigate whether the “island sign” pattern is associated with END.

**Methods:**

We enrolled consecutive patients admitted with acute PATI within 48 h after onset from May 2020 to July 2022. They were divided into with and without the “island sign” pattern on DWI. According to infarct location, all the patients were classified into two groups: the territories of the lenticulostriate arteries (LSA) and paramedian pontine arteries (PPA). The patients in each group were further divided into two groups according to whether they developed END or not. Through analyzing the clinical and neuroimaging characteristics of the patients, we tried to identify the factors that might associated with the “island sign” pattern and the potential predictors of END within the LSA and PPA groups.

**Results:**

Out of the 113 patients enrolled in this study, END was found in 17 patients (27.9%) in the LSA group and 20 patients (38.5%) in the PPA group. The “island sign” was found in 26 (23%) patients. In the multivariate analysis, the independent predictors of END in the LSA group were the “island sign” (OR 4.88 95% CI 1.03–23.2 *P* = 0.045) and high initial National Institute of Health Stroke Scale (NIHSS) (OR 1.79 95% CI 1.08–2.98 *P* = 0.024) and in the PPA group was the presence of lesions extending to the ventral pontine surface (OR 7.53 95% CI 1.75–32.37 *P* = 0.007).

**Conclusions:**

The predictive factors for END were different in the LSA and PPA groups. The “island sign” was particularly associated with END in the LSA group.

**Supplementary Information:**

The online version contains supplementary material available at 10.1186/s12883-023-03351-y.

## Introduction

Penetrating artery territory infarctions (PATIs) constitute up to 25% of all ischemic strokes, and patients generally have a better prognosis than patients with major brain artery occlusion [1]. However, 14%-40% of PATI patients are reported to develop early neurological deterioration (END) [1–4]. The worsening involves various neurological symptoms especially motor function and often result in severe disability [5]. Although several clinical factors have been associated with END, including female sex, initial systolic blood pressure, mean platelet volume, and high National Institute of Health Stroke Scale (NIHSS) score on admission [3, 6, 7], the mechanism and specific predictors remain unestablished [8].

Over the past decade, MRI findings such as lesion size and location on diffusion-weighted imaging (DWI) have been reported to be potential predictors of END in PATI [9, 10]. However, few studies have focused on the specific shape of the infarctions. It is crucial to discover the mechanisms underlying the infarct shape for developing appropriate treatment strategies. Most previous studies considered patients with isolated lesions; nevertheless, as observed in clinical practice, patients with multiple lesions in a PATI territory seem more likely to develop deterioration in the hospital. Therefore, we hypothesized that multiple lesions (represented as the “island sign”) on DWI would be a predictor of END. In the study of Yu and Tan [2], this multiple lesion pattern was named the “satellite lesion sign” to distinguish it from single oval lesions. In view of the irregular shape of the infarctions on DWI in many cases, we use the “island sign” instead of the “satellite lesion sign” to describe the pattern. It has been suggested in several studies that the risk factors are different in the territories of the lenticulostriate arteries (LSA) and paramedian pontine arteries (PPA) [6, 11]. The goal of this study was to analyze patients with PATI in the LSA and PPA territories to assess the predictors of END in the two groups of patients.

## Methods

### Patient selection

**Fig. 1:**
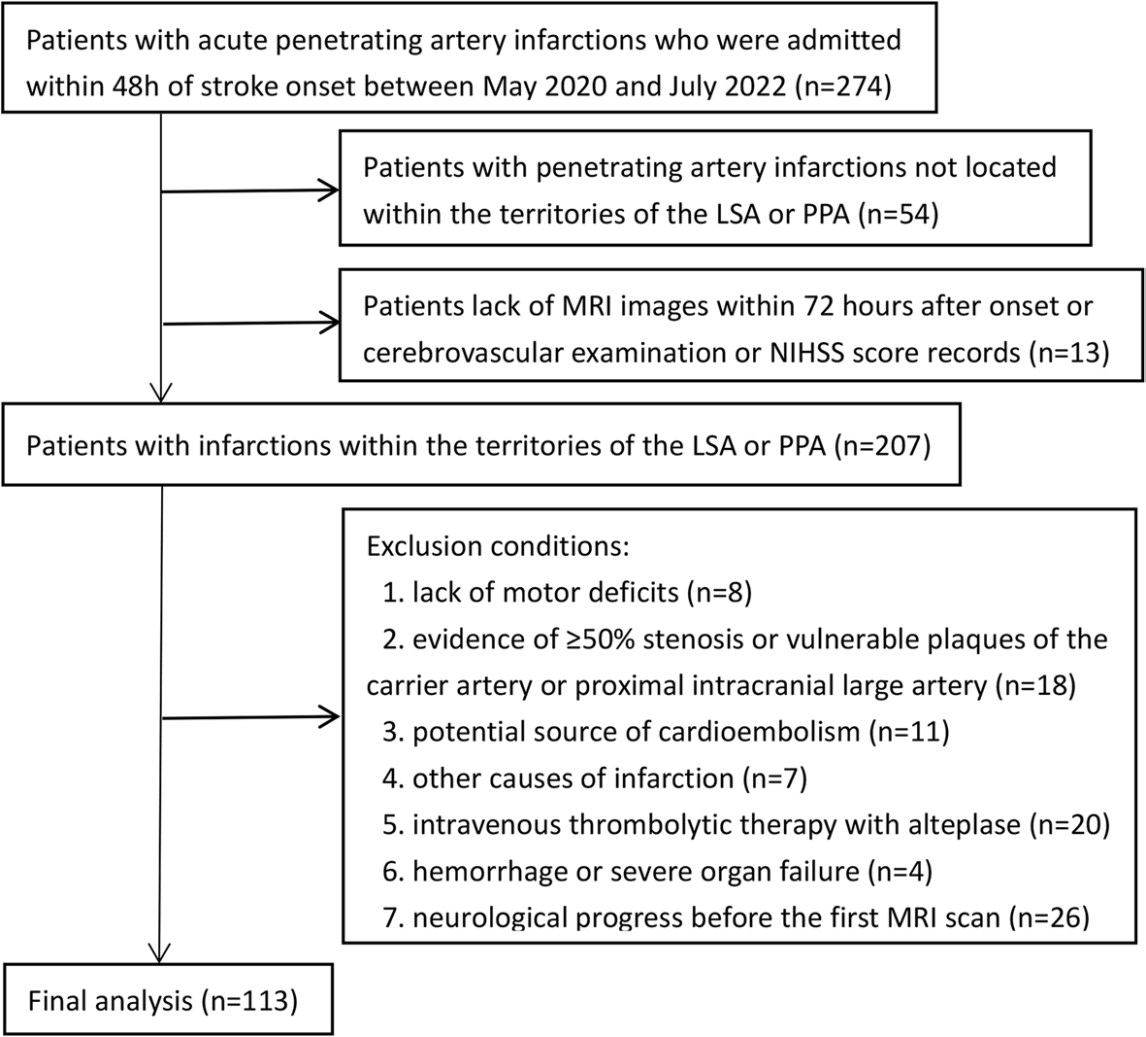
Patient flowchart. Abbreviations: LSA, lenticulostriate arteries; PPA, paramedian pontine arteries; NIHSS, national institute of health stroke scale

We retrospectively examined consecutive patients with acute ischemic stroke admitted to the First Affiliated Hospital of Shandong First Medical University from May 2020 to July 2022. Among them, patients who met the following criteria were enrolled: (1) admitted within 48 h of onset with a lacunar syndrome; (2) patients with penetrating artery territory infarctions confirmed by MRI-DWI; (3) the lacunar syndrome was attributed to the infarctions. Patients with the following conditions were excluded (Fig. 1): (1) infarctions not located within the territories of the LSA or PPA [12]; (2) lack of motor deficits (facial palsy, motor arm, motor leg, limb ataxia, or dysarthria); (3) evidence of ≥ 50% stenosis or vulnerable plaques of the carrier artery or proximal internal carotid and vertebral artery; (4) a potential source of cardioembolism (mitral stenosis, prosthetic heart valve, atrial fibrillation, recent myocardial infarction, infective endocarditis, etc.) [13]; (5) a history of hematological disorders, coagulopathy, malignant tumor, moyamoya disease, dissection or vasculitis of the intracranial carotid artery, middle cerebral artery and vertebrobasilar artery; (6) current treatment with intravenous thrombolysis therapy; (7) hemorrhage or severe organ failure during the observation period; (8) neurological progress before arrival or before the first MRI scan after arrival; (9) lack of MRI images within 72 h after onset or cerebrovascular examination or NIHSS score records. Acute medical treatments were performed following the current practice guidelines. All the patients were treated with antiplatelets (mono-antiplatelet or dual-antiplatelet therapy) and stain.

### Collection and definition of clinical data

We collected demographic and clinical data from the patients’ records, including age, sex, NIHSS scores on arrival and within 7 days, TIA history in the previous week and history of hypertension, diabetes mellitus, dyslipidemia, smoking and ischemic stroke. Hypertension and diabetes mellitus were defined according to the latest guidelines and standards [14, 15]. Dyslipidemia was defined as a total cholesterol (TC) level ≥ 6.2 mmol/l, low-density lipoprotein cholesterol (LDL-C) ≥ 2.6 mmol/l or triglyceride (TG) ≥ 1.8 mmol/l, or current treatment with lipid-lowing drugs [16]. Habitual smoking was defined as current or former regular smoking. Previous cerebral infarction or TIA was identified as an ischemic stroke history. We gathered laboratory information, including platelet count (PLTc), mean platelet volume (MPV), fibrinogen, D-dimer, TG, LDL-C and homocysteine, from the patients, all of whom were assessed within 24 h after admission. A twelve-lead ECG was performed for all patients.

### Evaluation of END

The evaluation of neurological deterioration was performed using the NIHSS score recorded on arrival and over the following 7 days 1–2 times daily by certified neurologists. END was defined as an increase in the NIHSS score ≥ 1 point in motor function or ≥ 2 points in total during the first 5 days after symptom onset [17]. The neurologic worsening should be persistent in order to distinguish from clinical fluctuations, whose deterioration was transient and reversed in the later course. If not, the patient was defined as non-END. The time from onset to END was collected.

### Evaluation of neuroimaging information

**Fig. 2:**
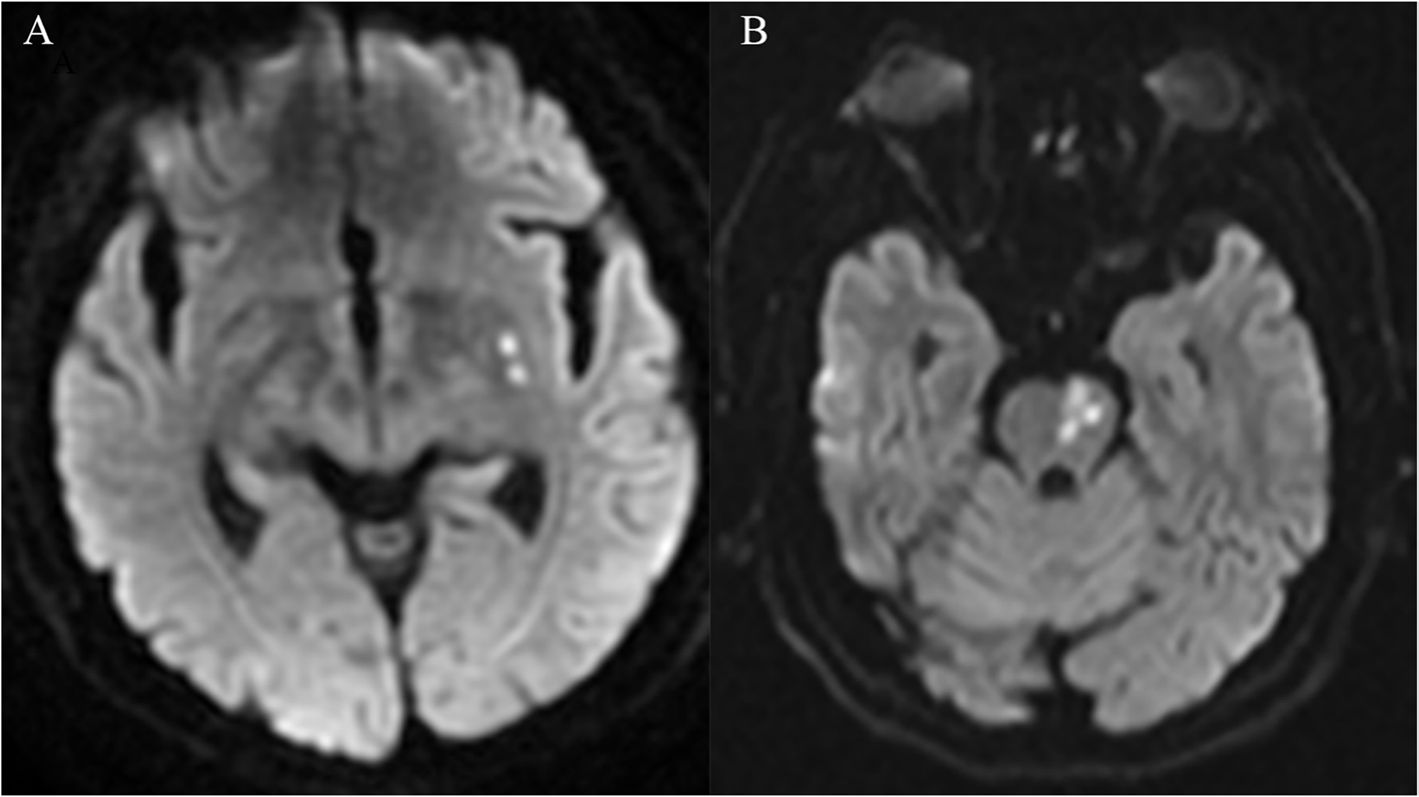
(A) Example of the “island sign”pattern on DWI in the lenticulostriate arteries (LSA) territory. (B) Example of the “island sign” pattern on DWI in the paramedian pontine arteries (PPA) territory

All participants underwent 3.0-T MRI (Siemens Skyra Vision) within 72 h after onset. Conventional spin‒echo sequences for cross-sectional T1-weighted (T1WI), T2-weighted (T2WI), and DWI were performed. “Infarct size” was measured as the maximal diameter of the lesions on the axial DWI scan (slice thickness 5 mm; interslice gap 1.5 mm; field of view 220 mm; repetition time 4240 ms; echo time 64 ms; matrix number 160 × 160; b value = 0; 1000 s/mm^2^). The “island sign” was defined as at least one independent lesion beside the main lesion on an axial DWI slice within the same penetrating artery territory of the LSA or PPA, regardless of shape and number (Fig. 2). The intracranial carotid arteries, middle cerebral arteries and vertebrobasilar arteries of all the patients were assessed by MRA or CTA within one week. The severity of stenosis in each relevant artery was graded based on the maximal luminal narrowing, as follows: normal, mild stenosis (≤ 50%) and moderate or severe stenosis (≥ 50%). Carrier artery stenosis was defined as the presence of mild stenosis at the middle cerebral artery or basilar artery within infarctions of the LSA or PPA territory, respectively. All neuroimaging data were evaluated by neuroradiologists who were blinded to the patients’ characteristics. The “island sign” was assessed by two neuroradiology specialists (*κ* values, 0.846), and a senior colleague judged any inconsistencies and made the final decision.

### Statistical analysis

Numerical variables (age, initial NIHSS, PLTc, MPV, etc.) are presented as the mean ± standard deviation or median (interquartile range). Categorical variables (sex, hypertension, smoking history, island sign, etc.) are reported as the percentage. Univariate comparisons of clinical characteristics were performed with Student’s *t* test (normally distributed variables), the Mann‒Whitney *U* test (nonnormally distributed variables), ***χ***^***2***^ test or Fisher’s exact test (categorical variables). Variables with a *P* value ≤ 0.1 in the univariate analysis were included in the multiple logistic regression models to determine the independent predictors of END in all the samples and subgroups. *P* < 0.05 was considered significant. SPSS version 26.0 software (Chicago, IL, USA) was used to perform all the statistical analyses.

## Results

### Baseline and demographic characteristics

A total of 113 consecutive inpatients diagnosed with acute cerebral infarction within the territories of the LSA and PPA (mean age ± SD = 65.4 ± 10.11 years; 44 females) were included in the final analysis. Of them, 85 (75.2%) patients had hypertension, 41 (36.3%) patients had diabetes mellitus, 38 (33.6%) patients had dyslipidemia, 43 (38.1%) patients had a smoking history, 25 (22.1%) patients had an ischemic stroke history, and 10 (8.8%) patients suffered a TIA in the week before onset. Overall, the median NIHSS score was 2.58 (IQR 1.62–3.74) on arrival, 61 (54%) patients had infarcts of the LSA territory, 26 (23%) showed the “island sign” on DWI, and 9 (8%) patients had carrier artery stenosis.

### Demographic and clinical characteristics between patients with and without the “island sign”

Of the 113 patients, the “island sign” was found in 26 (23%) patients. In the comparison of the characteristics between patients with and without the “island sign”, females (*P* = 0.025) were more likely to present with the pattern. In the LSA group, patients with an infarct size ≥ 15 mm (*P* = 0.003) were more prone to show the “island sign” than those without. The other demographic and clinical factors had no significant associations with the “island sign”, as shown in Table 1.

**Table 1:** Comparison of demographic and clinical characteristics between patients with and without the “island sign”

	With island sign	Without island sign	*P*
**All patients**	*N* = 26	*N* = 87	
Age, years, median(IQR)	65 (60.25, 73)	65 (57, 71)	0.728
Female sex, *N*%	15 (57.7)	29 (33.3)	0.025^*^
Hypertension, *N*%	19 (73.1)	66 (75.9)	0.773
Diabetes mellitus, *N*%	10 (38.5)	31 (35.6)	0.792
Dyslipidemia, *N*%	10 (38.5)	28 (32.2)	0.552
Ischemic stroke history, *N*%	5 (19.2)	20 (23.0)	0.685
TIA in the previous week, *N*%	2 (7.7)	8 (9.2)	1.000
Smoking history, *N*%	8 (30.8)	35 (40.2)	0.383
Initial NIHSS, median(IQR) Onset to initial MRI, h, mean ± SD	3 (1.75, 4) 33.87 ± 16.14	2 (2, 4) 31.96 ± 16.56	0.911 0.606
LSA territory, *N*%	14 (53.9)	47 (54.0)	0.987
Carrier artery stenosis, *N*%	4 (15.4)	5 (5.8)	0.238
**LSA**	*N* = 14	*N* = 47	
Initial NIHSS, median(IQR) Onset to initial MRI, h, mean ± SD	2.5 (1.75, 4) 30.07 ± 15.06	2 (2, 3) 27.89 ± 15.98	0.634 0.652
Carrier artery stenosis, *N*%	3 (21.4)	2 (4.3)	0.133
Infarct size ≥ 15 mm, *N*%	10 (71.4)	13 (27.7)	0.003^*^
Axial slices ≥ 3, *N*%	10 (71.4)	33 (70.2)	1.000
**PPA**	*N* = 12	*N* = 40	
Initial NIHSS, median(IQR) Onset to initial MRI, h, mean ± SD	3 (1.25, 3.75) 38.30 ± 16.86	3 (2, 4) 36.74 ± 16.14	0.444 0.773
Carrier artery stenosis, *N*%	1 (8.3)	3 (7.5)	1.000
Lesions extending to the ventral pontine surface, *N*%	9 (75.0)	21 (52.5)	0.116

*IQR* Interquartile range, *NIHSS* National institute of health stroke scale, *LSA* Lenticulostriate arteries, *PPA*, Paramedian pontine arteries

^*^P < 0.05

### Clinical, radiological and laboratory predictors of END

**Fig. 3:**
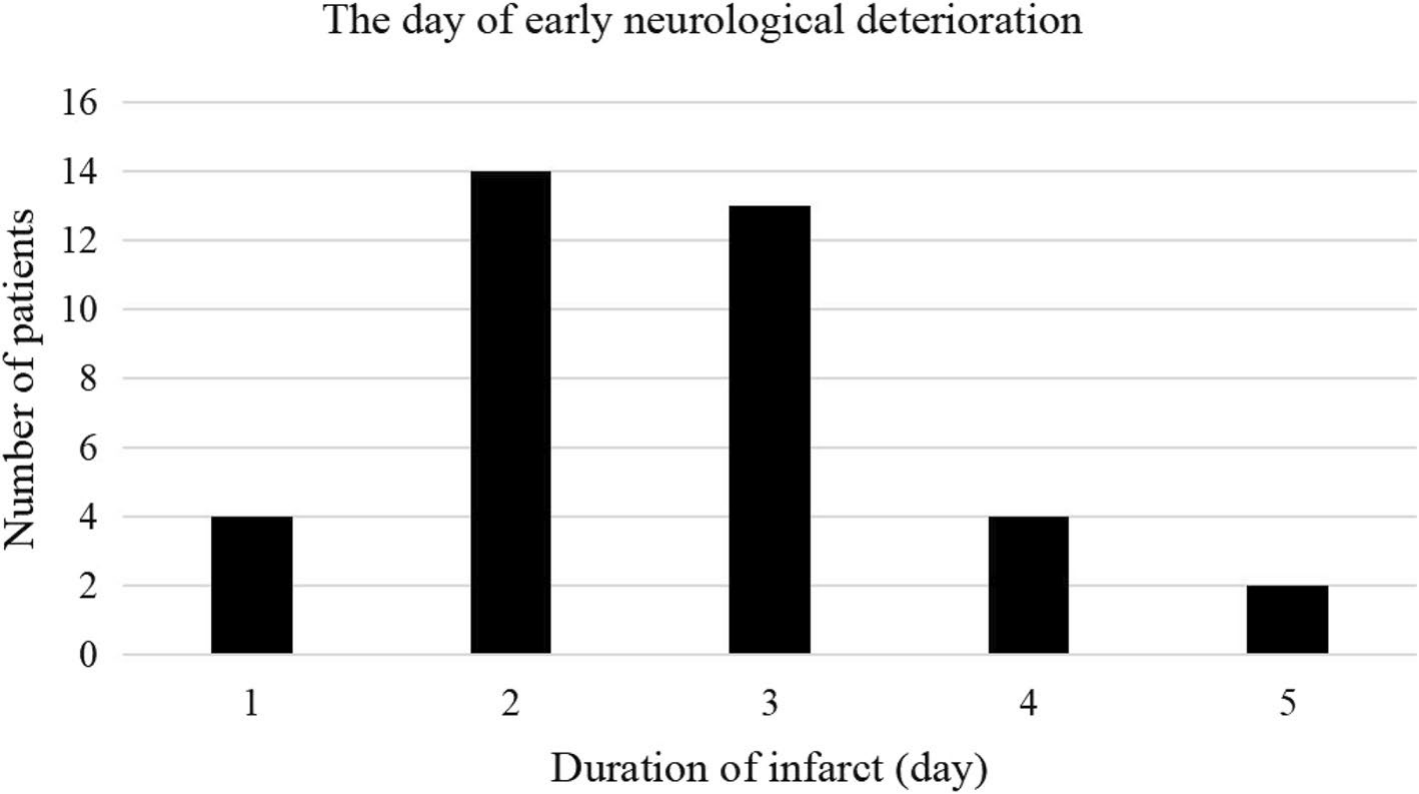
The day of END. The vertical line shows the number of patients with END and the horizontal line shows the duration of infarct. Thirty-seven patients (32.7%) exhibited END, 83.7% of the patients with END were gathered during the first 72 h after onset. Abbreviations: END, early neurological deterioration

Thirty-seven patients (32.7%) exhibited END, 48.6% of these patients had END within the first 48 h and 35.1% within the subsequent 24 h after onset (Fig. 3). The presence of the “island sign” was significantly different between the patients with and without END. After adjusting for all potential confounders (including smoking history, initial NIHSS, “island sign”, MPV, all of which had a *P* value ≤ 0.1 in the univariate analysis), the “island sign” (OR 4.86, 95% CI 1.79–13.19, P = 0.002) and a high initial NIHSS (OR 1.38 95% CI 1.05–1.82, P = 0.023) were identified as independent predictors for END (Table 2).

**Table 2:** Comparison of END and non-END in all the patients

Characteristics	END		*P*	Logistic regression		
	Yes(*N* = 37)	No(*N* = 76)		OR	95% CI	*P*
Age, years, mean ± SD	64.95 ± 9.13	65.62 ± 10.60	0.742			
Female, *N*%	16 (43.2)	28 (36.8)	0.513			
Hypertension, *N*%	25 (67.6)	60 (78.9)	0.189			
Diabetes mellitus, *N*%	14 (37.8)	27 (35.5)	0.810			
Dyslipidemia, *N*%	13 (35.1)	25 (32.9)	0.813			
Ischemic stroke history, *N*%	10 (27.0)	15 (19.7)	0.381			
TIA in the previous week, *N*%	6 (16.2)	4 (5.3)	0.116			
Smoking history, *N*%	10 (27.0)	33 (43.4)	0.092	0.55	(0.21 1.42)	0.216
Initial NIHSS, median(IQR)	3 (2, 4)	2 (2, 3)	0.064	1.38	(1.05 1.82)	0.023^*^
Dual-antiplatelet therapy, *N*%	29 (78.4)	57 (75.0)	0.693			
LSA territory, *N*%	17 (45.9)	44 (57.9)	0.232			
Island sign, *N*%	16 (43.2)	10 (13.2)	0.000	4.86	(1.79 13.19)	0.002^*^
Carrier artery stenosis, *N*%	5 (13.5)	4 (5.3)	0.25			
PLTc, *10^9/L, median(IQR)^a^	214.5 (185.75, 256.75)	224.5 (190, 267.5)	0.418			
MPV, fL, median(IQR)^a^	10.35 (9.72, 10.90)	9.90 (9.50, 10.68)	0.069	1.33	(0.85 2.08)	0.212
Fibrinogen, g/L, mean ± SD^a^	2.67 ± 0.64	2.91 ± 0.77	0.115			
D-dimer, mg/L, median(IQR)^a^	0.27 (0.17, 0.49)	0.28 (0.21, 0.59)	0.391			
TG, mmol/L, median(IQR)	1.06 (0.91, 1.98)	1.17 (0.87, 1.80)	0.653			
LDL-C, mmol/L, mean ± SD	2.63 ± 0.68	2.58 ± 0.72	0.711			
Hcy, umol/L, median(IQR)^a^	13.30 (10.25, 14.40)	12.80 (11.30, 16.70)	0.428			

^a^PLTc, MPV, Fibrinogen, D-dimer and Hcy data of 1 patient was unavailable

*END* Early neurological deterioration, *NIHSS* National institute of health stroke scale, *LSA* Lenticulostriate arteries, *PLTc* Platelet count, *MPV* Mean platelet volume, *TG* Triglyceride, *LDL-C* Low-density lipoprotein cholesterol, *Hcy* Homocysteine, *OR* Odds ratio, *CI* Confidence interval, *SD* Standard deviation, *IQR* Interquartile range

^*^P < 0.05

### Factors of END in the LSA group and PPA group

The rate of END was 27.9% in the LSA group and 38.5% in the PPA group. Multivariate logistic regression analysis with the variables with *P* ≤ 0.1 in the univariate analysis revealed that the “island sign” (OR 4.88 95% CI 1.03–23.02 *P* = 0.045) and a high initial NIHSS (OR 1.79 95% CI 1.08–2.98 *P* = 0.024) in the LSA group and lesions extending to the ventral pontine surface (OR 7.53 95% CI 1.75–32.37 *P* = 0.007) in the PPA group remained independent predictive factors for END, as shown in Table 3.

**Table 3:** Comparison of END and non-END in the LSA group and PPA group

	END		*P*	Logistic regression		
	Yes	No		OR	95% CI	*P*
**LSA**	*N* = 17	*N* = 44				
Age, years, mean ± SD	66.71 ± 10.20	64.84 ± 11.58	0.563			
Female, *N*%	8 (47.1)	17 (38.6)	0.549			
Hypertension, *N*%	12 (70.6)	34 (77.3)	0.832			
Diabetes mellitus, *N*%	4 (23.5)	10 (22.7)	1.000			
Dyslipidemia, *N*%	5 (29.4)	11 (25.0)	0.979			
Ischemic stroke history, *N*%	4 (23.5)	8 (18.2)	0.911			
TIA in the previous week, *N*%	2 (4.6)	3 (6.8)	0.912			
Smoking history, *N*%	5 (29.4)	18 (40.9)	0.406			
Initial NIHSS, median(IQR) Dual-antiplatelet therapy, *N*%	4 (2, 5) 11 (64.7)	2 (2, 3) 33 (75.0)	0.009 0.627	1.79	(1.08 2.98)	0.024^*^
Island sign, *N*%	8 (47.1)	6 (13.6)	0.015	4.88	(1.03 23.02)	0.045^*^
Carrier artery stenosis, *N*%	3 (17.7)	2 (4.6)	0.249			
PLTc, *10^9/L, median(IQR)^a^	209.5 (179, 240.75)	232 (217.5, 282.25)	0.035	0.99	(0.97 1.00)	0.106
MPV, fL, mean ± SD^a^	10.49 ± 1.47	9.93 ± 0.77	0.169			
D-dimer, mg/L, median(IQR)	0.32 (0.25, 0.65)	0.29 (0.21, 0.58)	0.676			
Fibrinogen, g/L, median(IQR)	2.65 (2.18, 3.02)	2.87 (2.52, 3.30)	0.177			
TG, mmol/L, median(IQR)	1.03 (0.83, 1.37)	1.00 (0.78, 1.45)	0.816			
LDL-C, mmol/L, mean ± SD	2.68 ± 0.64	2.67 ± 0.64	0.953			
Hcy, umol/L, median(IQR)	12.30 (8.55, 14.20)	13.70 (11.68, 17.50)	0.048	0.87	(0.71 1.06)	0.176
Infarct size ≥ 15 mm, *N*%	11 (64.7)	12 (27.3)	0.007	1.19	(0.25 5.61)	0.824
**PPA**	N = 20	N = 32				
Age, years, mean ± SD	63.45 ± 8.08	66.69 ± 9.16	0.201			
Female, *N*%	8 (40.0)	11 (34.4)	0.682			
Hypertension, *N*%	13 (65.0)	26 (81.3)	0.188			
Diabetes mellitus, *N*%	10 (50.0)	17 (53.1)	0.826			
Dyslipidemia, *N*%	8 (40.0)	14 (43.8)	0.790			
Ischemic stroke history, *N*%	6 (30.0)	7 (21.9)	0.510			
TIA in the previous week, *N*%	4 (20.0)	1 (3.1)	0.127			
Smoking history, *N*%	5 (25.0)	15 (46.9)	0.115			
Initial NIHSS, median(IQR) Dual-antiplatelet therapy, *N*%	3 (2, 4) 18 (90.0)	3 (2, 4) 24 (75.0)	0.737 0.330			
Island sign, *N*%	8 (40.0)	4 (12.5)	0.051	3.96	(0.87 18.06)	0.075
Carrier artery stenosis, *N*%	2 (10.0)	2 (6.3)	1.000			
PLTc, *10^9/L, mean ± SD	230.60 ± 53.64	211.13 ± 46.02	0.170			
MPV, fL, median(IQR)	10.35 (9.93, 10.90)	10.00 (9.63, 10.78)	0.254			
D-dimer, mg/L, median(IQR)^a^	0.24 (0.16, 0.42)	0.28 (0.22, 0.62)	0.109			
Fibrinogen, g/L, mean ± SD^a^	2.76 ± 0.53	2.91 ± 0.65	0.388			
TG, mmol/L, median(IQR)	1.37 (0.97, 2.23)	1.39 (1.04, 2.21)	0.962			
LDL-C, mmol/L, mean ± SD	2.59 ± 0.72	2.46 ± 0.81	0.544			
Hcy, umol/L, median(IQR)^a^	13.80 (11.35, 14.55)	12.50 (10.60, 16.10)	0.364			
Lesions extending to the ventral pontine surface, *N*%	17 (85.0)	13 (40.6)	0.002	7.53	(1.75 32.37)	0.007^*^

^a^PLTc, MPV, Fibrinogen, D-dimer and Hcy data of 1 patient was unavailable

*END* Early neurological deterioration, *LSA* Lenticulostriate arteries, *NIHSS* National institute of health stroke scale, *PLTc* Platelet count, *MPV* Mean platelet volume, *TG* Triglyceride, *LDL-C* Low-density lipoprotein cholesterol, *Hcy* Homocysteine, *PPA* paramedian pontine arteries, *OR* Odds ratio, *CI* Confidence interval, *SD* Standard deviation, *IQR* Interquartile range

^*^P < 0.05

## Discussion

In our population, END occurred in 32.7% of all patients: 27.9% of the LSA group and 38.5% of the PPA group. Here, the DWI “island sign” was found to be an independent predictor for END in penetrating artery territory infarctions and in the LSA group. However, in the PPA group, lesions extending to the ventral pontine surface were observed to be an independent risk factor for END. To the best of our knowledge, few studies have focused on the relationship between infarction shape in the perforating arterial territory and END, especially in the territories of the LSA and PPA.

In previous studies, branch atheromatous disease (BAD) was revealed to be associated with progressive neurological deficits [18]; this condition is reported to result from occlusion or stenosis at the origin of a deep penetrating artery [19] and lead to large ischemic lesions [20]. Nakase, Yoshioka et at. [9] defined BAD as an intracerebral lesion ≥ 15 mm in diameter spanning more than 3 slices or a lesion extending to the surface of the pontine base on DWI; this definition has been applied in several studies [7, 11, 16, 21]. In our study, patients with lesions extending to the ventral pontine surface tended to develop END in the PPA group, which is consistent with the aforementioned studies. However, the infarct size was not independently corelated with END in the LSA group. This might be attributed to the different stages of DWI examination in the present study and the previous studies [9, 21] and the restriction of our population to those prior to neurological deterioration. Nevertheless, we found that the proportion of patients with “infarct size ≥ 15 mm” in the END group was higher than that in the non-END group. This might be more relevant to the multiple-lesion shape in the END group because of the increased “infarct size”. Thus, we speculated that if the DWI was evaluated at a different stage or after neurological deterioration, early small lesions could be enlarged, and multiple lesions can be integrated into large-sized infarcts, which was estimated as a predictive factor of END.

In this study, we discovered that the “island sign” on DWI was independently associated with END, especially in the LSA group. Additionally, we found that there were more lesions with a maximal diameter ≥ 15 mm in the “island sign” group than in the group without the “island sign”. Larger ischemic lesions are said to be the result of vascular lesions located proximally along the perforator artery [22], which are usually caused by junctional plaques or microatheroma within the orifice of the penetrating artery [23]. Studies based on high-resolution magnetic resonance imaging (HR-MRI) have shown that plaques on the middle cerebral artery are related to larger infarction lesions in the LSA region and END [24, 25]. Very recently, studies using perfusion MRI have suggested that hemodynamic compromise and lack of collaterals in PATI play important roles in the development of END [26, 27]. We speculated that the possible mechanisms of the “island sign” in the perforating arterial territory might be 1) extension of the atheromatous plaques at the orifice of the penetrating arteries or the parent arteries that leads to occlusion at more than one branch of the perforating artery; 2) stenosis at the origin of the branches and the absence of collateral vessels, leading to high susceptibility to hypoperfusion, and mimicking of watershed infarcts by the subsequent infarction [23, 28]; 3) dropping off of the unstable plaque in the parent artery that results in the embolization of several penetrating arteries; or 4) the contribution of multiple mechanisms. A territory of more than one branch was involved in the suspected pathogenesis mentioned above. Progression of thrombosis and embolism may cause more involvement and stepwise perfusion reduction of branches, which ultimately contribute to END. In addition, other mechanisms, such as edema, excitotoxicity and inflammation, may also be involved in the process [5]. As defined before, the “island sign” was observed on axial DWI scans and is approximately vertical to the lenticulostriate arteries; thus, it was easy to discover lesions caused by multiple branches. However, the axial section was nearly parallel to most of the basilar branches, which may explain why the “island sign” was not independently associated with END in the PPA territory.

In our study, 22 (nearly 60%) patients of the END group performed repeat CT or MRI after the neurological deterioration, recurrent infarct lesion was not found within them. In previous study, the cumulative rate of the first recurrent ischemic stroke at 7 days was 1.4% [29] and most of the primary infarcts are classified into “large artery atherosclerosis” and “cardioembolism” according to the TOAST criteria [30]. However, in this study, almost 80% of included patients belonged to the subtype of “small artery occlusion”. Therefore, we suspected that recurrent stroke in the included patients during the first week was very rare and might have little effect on the final results.

There were several limitations to our study. First, this was a retrospective study in a single center involving a small sample size, and the results need to be further confirmed by multicenter prospective studies with large samples. Second, because of some practical difficulties, repeat MRI was not performed in all the patients, and the aforementioned suspected enlargement of lesions was not observed on DWI. Third, the “island sign” was defined on an axial DWI scan, and other images, such as coronal scans, were not available. However, we hypothesize that the presence of the “island sign” on coronal DWI may be related to END in the PPA territory.

## Conclusion

In summary, our study indicated that the presence of the “island sign” on DWI may independently predict END in patients with LSA territory infarction. The underlying mechanisms need to be explored by further investigations. According to recent studies, END was found to be associated with unfavorable functional outcomes at 3 months [31]. Therefore, we suggested that neurological physicians should pay more attention to patients with this special form of infarct shape and implement more aggressive and individualized therapy in the early stage to prevent END.

### Supplementary Information

**Additional file 1. **Supplementary material: All participants underwent a 24-hours electrocardiogram monitoring routinely after admission. Among the 26 patients with the “island sign”, echocardiography was performed in 23 (88.5%) patients during the hospitalization and in 3 (11.5%) patients within 1 month after discharge.

## Data Availability

The datasets used and/or analysed during the current study are available from the corresponding author on reasonable request.

## Abbreviations

PATI: Penetrating artery territory infarction
END: Early neurological deterioration
NIHSS: National Institute of Health Stroke Scale
DWI: Diffusion-weighted imaging
LSA: Lenticulostriate arteries
PPA: Paramedian pontine arteries
TC: Total cholesterol
LDL-C: Low-density lipoprotein cholesterol
TG: Triglyceride
PLTc: Platelet count
MPV: Mean platelet volume
T1WI: T1-weighted
T2WI: T2-weighted
BAD: Branch atheromatous disease
HR-MRI: High-resolution magnetic resonance imaging

## References

[1] Kawano T, Miyashita K, Takeuchi M, Nagakane Y, Yamamoto Y, Kamiyama K (2018). Blood biomarkers associated with neurological deterioration in patients with acute penetrating artery territory infarction: A multicenter prospective observational study. Int J Stroke.

[2] Yu Y-P, Tan L (2015). The Infarct Shape Predicts Progressive Motor Deficits in Patients with Acute Lacunae-sized Infarctions in the Perforating Arterial Territory. Intern Med.

[3] Takeuchi M, Miyashita K, Nakagawara J, Toyoda K, Todo K, Metoki N (2016). Analysis of Factors Associated with Progression and Long-Term Outcomes of Penetrating Artery Territory Infarction: A Retrospective Study. J Stroke Cerebrovasc Dis.

[4] Gong P, Liu Y, Huang T, Chen W, Jiang T, Gong Y (2019). The association between high-sensitivity C-reactive protein at admission and progressive motor deficits in patients with penetrating artery infarctions. BMC Neurol.

[5] Del Bene A, Palumbo V, Lamassa M, Saia V, Piccardi B, Inzitari D (2012). Progressive lacunar stroke: review of mechanisms, prognostic features, and putative treatments. Int J Stroke.

[6] Yamamoto Y, Ohara T, Hamanaka M, Hosomi A, Tamura A, Akiguchi I (2010). Predictive factors for progressive motor deficits in penetrating artery infarctions in two different arterial territories. J Neurol Sci.

[7] Oji S, Tomohisa D, Hara W, Tajima T, Suzuki M, Saito A (2018). Mean Platelet Volume Is Associated with Early Neurological Deterioration in Patients with Branch Atheromatous Disease: Involvement of Platelet Activation. J Stroke Cerebrovasc Dis.

[8] Petrone L, Nannoni S, Del Bene A, Palumbo V, Inzitari D (2016). Branch Atheromatous Disease: A Clinically Meaningful. Yet Unproven Concept Cerebrovasc Dis.

[9] Nakase T, Yoshioka S, Sasaki M, Suzuki A (2013). Clinical evaluation of lacunar infarction and branch atheromatous disease. J Stroke Cerebrovasc Dis.

[10] Duan Z, Fu C, Chen B, Xu G, Tao L, Tang T (2015). Lesion patterns of single small subcortical infarct and its association with early neurological deterioration. Neurol Sci.

[11] Takahashi Y, Yamashita T, Morihara R, Nakano Y, Sato K, Takemoto M (2017). Different Characteristics of Anterior and Posterior Branch Atheromatous Diseases with or without Early Neurologic Deterioration. J Stroke Cerebrovasc Dis.

[12] Tatu L, Moulin T, Vuillier F, Bogousslavsky J (2012). Arterial territories of the human brain. Front Neurol Neurosci.

[13] Gao S, Wang YJ, Xu AD, Li YS, Wang DZ (2011). Chinese ischemic stroke subclassification Front Neurol.

[14] Unger T, Borghi C, Charchar F, Khan NA, Poulter NR, Prabhakaran D (2020). 2020 International Society of Hypertension Global Hypertension Practice Guidelines. Hypertension.

[15] American Diabetes A. 2. Classification and Diagnosis of Diabetes: Standards of Medical Care in Diabetes-2021. Diabetes Care. 2021;44(Suppl 1):S15–33. 10.2337/dc21-S002 33298413

[16] Men X, Li J, Zhang B, Zhang L, Li H, Lu Z (2013). Homocysteine and C-reactive protein associated with progression and prognosis of intracranial branch atheromatous disease. PLoS ONE.

[17] Ohara T, Yamamoto Y, Tamura A, Ishii R, Murai T (2010). The infarct location predicts progressive motor deficits in patients with acute lacunar infarction in the lenticulostriate artery territory. J Neurol Sci.

[18] Yamamoto Y, Ohara T, Hamanaka M, Hosomi A, Tamura A, Akiguchi I (2011). Characteristics of intracranial branch atheromatous disease and its association with progressive motor deficits. J Neurol Sci.

[19] Fisher CM, Caplan LR (1971). Basilar artery branch occlusion: a cause of pontine infarction. Neurology.

[20] Duan Z, Sun W, Liu W, Xiao L, Huang Z, Cao L (2015). Acute diffusion-weighted imaging lesion patterns predict progressive small subcortical infarct in the perforator territory of the middle cerebral artery. Int J Stroke.

[21] Nakase T, Yamamoto Y, Takagi M (2015). Japan Branch Atheromatous Disease Registry C. The Impact of Diagnosing Branch Atheromatous Disease for Predicting Prognosis. J Stroke Cerebrovasc Dis.

[22] Nah HW, Kang DW, Kwon SU, Kim JS (2010). Diversity of single small subcortical infarctions according to infarct location and parent artery disease: analysis of indicators for small vessel disease and atherosclerosis. Stroke.

[23] Caplan LR (1989). Intracranial branch atheromatous disease: a neglected, understudied, and underused concept. Neurology.

[24] Sun LL, Li ZH, Tang WX, Liu L, Chang FY, Zhang XB (2018). High resolution magnetic resonance imaging in pathogenesis diagnosis of single lenticulostriate infarction with nonstenotic middle cerebral artery, a retrospective study. BMC Neurol.

[25] Yan Y, Jiang S, Yang T, Yuan Y, Wang C, Deng Q (2023). Lenticulostriate artery length and middle cerebral artery plaque as predictors of early neurological deterioration in single subcortical infarction. Int J Stroke.

[26] Pan Y-T, Tsai Y-H, Lee J-D, Weng H-H, Yang J-T, Huang Y-C. Evaluation of clinical relevance and underlying pathology for hemodynamic compromise in acute small subcortical infarction using MRI-based neuroimaging markers. Biomedical Journal. 2022. (in press) 10.1016/j.bj.2022.03.014 PMC10267958 35367449

[27] Huang YC, Lee JD, Pan YT, Weng HH, Yang JT, Lin LC (2022). Perfusion Defects and Collateral Flow Patterns in Acute Small Subcortical Infarction: a 4D Dynamic MRI Study. Transl Stroke Res.

[28] D'Amore C, Paciaroni M (2012). Border-zone and watershed infarctions. Front Neurol Neurosci.

[29] Kang K, Park TH, Kim N, Jang MU, Park SS, Park JM (2016). Recurrent Stroke, Myocardial Infarction, and Major Vascular Events during the First Year after Acute Ischemic Stroke: The Multicenter Prospective Observational Study about Recurrence and Its Determinants after Acute Ischemic Stroke I. J Stroke Cerebrovasc Dis.

[30] Saber H, Thrift AG, Kapral MK, Shoamanesh A, Amiri A, Farzadfard MT (2017). Incidence, recurrence, and long-term survival of ischemic stroke subtypes: A population-based study in the Middle East. Int J Stroke.

[31] Vynckier J, Maamari B, Grunder L, Goeldlin MB, Meinel TR, Kaesmacher J, et al. Early Neurologic Deterioration in Lacunar Stroke: Clinical and Imaging Predictors and Association With Long-term Outcome. Neurology. 2021. 10.1212/WNL.0000000000012661. 10.1212/WNL.0000000000012661 34400585

