# Supplementary material for: The “island sign” on diffusion-weighted imaging predicts early neurological deterioration in penetrating artery territory infarctions: a retrospective study
Source: BMC Neurol. 2023 Aug 11;23:298. doi: 10.1186/s12883-023-03351-y (PMC10416470; doi:10.1186/s12883-023-03351-y)
Supplement: Supplementary file 1 — Additional file 1. Supplementary material: All participants underwent a 24-hours electrocardiogram monitoring routinely after admission. Among the 26 patients with the “island sign”, echocardiography was performed in 23 (88.5%) patients during the hospitalization and in 3 (11.5%) patients within 1 month after discharge. [file 12883_2023_3351_MOESM1_ESM.docx]

Supplementary material:

All participants underwent a 24-hours electrocardiogram monitoring routinely after admission. Among the 26 patients with the “island sign”, echocardiography was performed in 23 (88.5%) patients during the hospitalization and in 3 (11.5%) patients within 1 month after discharge.
